# CoGNIT Automated Tablet Computer Cognitive Testing in Patients With Mild Cognitive Impairment: Feasibility Study

**DOI:** 10.2196/23589

**Published:** 2022-03-11

**Authors:** Anders Behrens, Johan Sanmartin Berglund, Peter Anderberg

**Affiliations:** 1 Department of Health Blekinge Institute of Technology Karlskrona Sweden

**Keywords:** internet, cognitive testing, software, testing, impairment, cognition, feasibility, diagnosis, app, assessment, cognitive impairment

## Abstract

**Background:**

Early diagnosis of cognitive disorders is becoming increasingly important. Limited resources for specialist assessment and an increasing demographical challenge warrants the need for efficient methods of evaluation. In response, CoGNIT, a tablet app for automatic, standardized, and efficient assessment of cognitive function, was developed. Included tests span the cognitive domains regarded as important for assessment in a general memory clinic (memory, language, psychomotor speed, executive function, attention, visuospatial ability, manual dexterity, and symptoms of depression).

**Objective:**

The aim of this study was to assess the feasibility of automatic cognitive testing with CoGNIT in older patients with symptoms of mild cognitive impairment (MCI).

**Methods:**

Patients older than 55 years with symptoms of MCI (n=36) were recruited at the research clinic at the Blekinge Institute of Technology (BTH), Karlskrona, Sweden. A research nurse administered the Mini-Mental State Exam (MMSE) and the CoGNIT app on a tablet computer. Technical and testing issues were documented.

**Results:**

The test battery was completed by all 36 patients. One test, the four-finger–tapping test, was performed incorrectly by 42% of the patients. Issues regarding clarity of instructions were found in 2 tests (block design test and the one finger-tapping test). Minor software bugs were identified.

**Conclusions:**

The overall feasibility of automatic cognitive testing with the CoGNIT app in patients with symptoms of MCI was good. The study highlighted tests that did not function optimally. The four-finger–tapping test will be discarded, and minor improvements to the software will be added before further studies and deployment in the clinic.

## Introduction

The global burden of dementia is estimated to increase from 43-47 million in 2016 to over 100 million patients by 2050 [1]. Early diagnosis of cognitive diseases is of increasing importance to assure optimal and early care, prognostics, and treatment. This is highlighted by the development of criteria for mild cognitive impairment (MCI), in which diagnostic criteria exist today for predementia stages of Alzheimer disease, dementia in Parkinson disease, and vascular cognitive impairment. Early detection might be even more important in the future when more potent treatments might exist that are likely to be more effective at an early stage of disease.

In the latest revision of the American Academy of Neurology practice guidelines for MCI (September 2019), yearly cognitive screening in healthy adults above 65 years old is encouraged [2]. Longitudinal assessments of patients with MCI are even more important, as approximately 50% might have a cognitive disease with progressive cognitive decline, and many patients revert to normal cognitive functioning [3,4]. This predicts an increasing demand for cognitive testing. Commonly used screening instruments such as the Mini-Mental State Exam (MMSE) are rapid but crude, and measurement error is larger than the typical yearly decline in Alzheimer disease [5,6]. Also, the utility for differential diagnostics is limited. Neuropsychological assessment with sensitive measures of several cognitive domains may aid diagnostics when basal investigation including screening instruments are inconclusive.

Today, less than one-third of patients in Sweden referred from primary to specialist care centers undergo a neuropsychological investigation. Given the demographical challenge, this portion is likely to decrease further. In response, CoGNIT, a computerized neuropsychological test battery, was developed, initially for assessing hydrocephalus patients. Tests are automatically delivered and scored on a tablet computer, assuring portability, standardization, and efficiency. Made for repeated assessments, included tests are chosen to be able to capture performance on a spectrum, rather than dichotomous variables, thus being able to pick up nuances in cognitive change. The test battery includes tests to cover several cognitive domains (memory, visuospatial function, attention, psychomotor speed, executive function, and manual dexterity). A summary score is calculated for all tests, making it possible to track changes in specific domains as well as global functioning over time.

A previous version of CoGNIT that ran on a touch-screen computer has been validated, norms and reliability data have been collected, and the test has been used in clinical practice in the management of hydrocephalus patients for over 5 years [7,8]. A novel version of CoGNIT running on a tablet has been expanded with tests of language, visuospatial ability, and upper extremity motor speed, to cover the cognitive domains of a typical evaluation in a memory clinic. The intended use cases are (1) as part of the investigation at a memory clinic for early detection of cognitive impairment; (2) in differential diagnostics, since diseases have different patterns of cognitive impairment; and (3) in follow-up testing after initiation of treatment (eg, acetylcholinesterase inhibitor) to assess the treatment effect.

The aim of this study was to assess the feasibility of automatic testing with CoGNIT in older persons with MCI. Data will be used to adapt the software for better performance before collecting normative data in a healthy population.

## Methods

### Ethical Approval

Ethical approval for this study was granted by the regional ethical review board in Lund, Sweden (dnr 2016/470).

### Recruitment

Patients with symptoms of MCI were recruited from the ongoing SMART4MD project at the research clinic at the Blekinge Institute of Technology (BTH), Karlskrona, Sweden [9]. This project evaluates a digital platform that supports patients with reminders, information, and memory support in everyday life. Patients in this project have several visits to the research clinic during their follow-up and were recruited for cognitive testing with CoGNIT during their second visit. The examiner (a research nurse) explained the experiment and collected signed consent from each subject. Patients were included in February 2020 and March 2020. Inclusion criteria were (data collected at first visit) MMSE of 20 to 28 points; subjective memory problems for >6 months; older than 55 years; participants in charge over their own medication; and no specific conditions reducing their ability to use an app, such as visual, hearing, or motor impairments.

Exclusion criteria were a terminal illness with less than 3 years of expected survival, Geriatric Depression Scale (GDS) score above 11, or cognitive impairment due to a known condition such as abuse or psychiatric illness.

### Procedures

The MMSE was administered to participants by the research nurse (the examiner). In direct succession, the CoGNIT computerized neuropsychological test battery was administered. While testing, the examiner was sitting by, answering questions, and taking notes regarding technical or testing issues. Patients were also asked to answer the TechPH questionnaire, a short instrument assessing older people’s attitude towards technology [10].

### CoGNIT Test Battery

#### Test Presentation and Instructions

Tests were presented on a 10.5” tablet computer (Samsung Galaxy Tab S6, 6 GB RAM, 128 GB internal storage; Samsung Electronics, South Korea) running the Android 11.0 operating system. The tablet was connected via Wi-Fi to the internet. CoGNIT was accessed on the Chrome web browser via an online server. Results from testing were stored in a database on the server for access via a web interface. Test instructions are presented with animations and sounds via a speaker. A trial round with automatic feedback precedes all tests. Two tests require verbal input from the patient that is recorded by the tablet microphone (10-word list test and category fluency test). These tests are manually corrected by the examiner after the testing session (simple procedure by “checking boxes” in the software). All other tests are automatically scored by the software. When scoring is completed, a pdf test report is automatically produced. Included tests are described in the following sections.

#### Memory

In the 10-word list test, 10 nouns are consecutively presented via text and sound. The patient is asked to say the remembered words aloud after presentation. The test is repeated for 3 trials. The sound is recorded for later manual scoring of the test. After approximately 15 minutes and 2 intervening distractor tasks, a free recall test is performed, without prior presentation of the words. In direct succession, a recognition test is performed. The patient is asked to press yes/no buttons indicating if the word was included in the learning test. A total of 20 words is presented: 10 correct and 10 distractor words.

#### Executive Function and Psychomotor Speed

The Stroop test consists of 2 parts, the Stroop congruent test and Stroop incongruent test. In the Stroop congruent test, the patient is asked to press 1 of 2 colored buttons on the screen indicated by text. In the Stroop incongruent test, the text is colored, and the patient is asked to press the button indicated by the color of the text and not what is written. Each trial includes 50 color-words. Response time and errors are collected.

Similarly, the Trail Making Test also includes 2 tests. In Trail Making Test A, the numbers 1 to 25 are presented on the screen, and the patient is asked to press each number in numerical order. In Trail Making Test B, both numbers (1-13) and letters (A-L) are presented on the screen. The patient is asked to press letters and number in order by alternating between successive numbers and letters of the alphabet (ie, 1-A-2-B-3-C...). Time to completion and errors are collected.

#### Attention

In the 2-choice reaction test, the patient is asked to press 1 of 2 buttons indicated by an arrow. The arrow appears after a random interval of 5 seconds to 15 seconds. Reaction time is measured for 20 trials.

#### Language

In the category fluency test, the patient is asked to say aloud as many words from a category (eg, animals) as possible for 1 minute. Sound is recorded and corrected by the examiner after all tests are completed.

#### Motor Speed

In the four-finger–tapping test, the patient is required to tap on a small keyboard with the digits 2 through 5. The correct order of taps is (digits) 2-3-4-5-4-3-2-3... The number of correct taps over 5 trials is scored. In the one-finger–tapping test, the patient taps 1 finger repeatedly between 2 circles on the screen. The number of taps during a 10-second period is recorded for 3 trials for both the left and right hands.

#### Visuospatial Ability

The block design test, a figure composed of 4 colored blocks is presented on the screen. The patient is asked to rearrange blocks presented on the top of the screen to match the pattern. Time to completion is scored for 3 different patterns.

#### Depression

Symptoms of depression were screened using the GDS, in which 15 questions are presented on the screen and the patient is required to answer by pressing buttons marked yes or no.

### Statistical Analysis

The completion rate without testing issues in patients was previously estimated at 80% in hydrocephalus patients. With a sample size of 36, an 80% issue-free completion rate can be estimated with a 95% CI of +/-13%, which was regarded as sufficient. This also assured for detecting issues for improvement. Influences on test completion by age and score on the TechPH and MMSE scales were assessed with Mann-Whitney *U* tests. Influence by gender and education was assessed with chi-squared tests. Significance level was set at *P*<.05. All statistics were analyzed in SPSS (Version 25; IBM Corp, Armonk, NY).

## Results

### Sample Characteristics

Of 42 patients, 36 (86%) agreed to participate; 4 patients declined participation due to a lack of interest or time, and 2 patients declined because of medical reasons (hemiparesis and severe visual impairment). Demographics for the study population are presented in Table 1. The age was rather high (mean 75.6 years). There was a slight overrepresentation of men (22/36, 61%).

**Table 1:** Demographic data for the study population.

Characteristics			Results
Age, (years), mean (SD)			75.6 (5.0)
**Sex, n (%)**			
	Male	22 (61)	
	Female	14 (39)	
**Education, n (%)**			
	Low	9 (25)	
	Medium	14 (39)	
	High	13 (36)	
MMSE^a^ (points), mean (SD)			28.2 (2.1)
TechPH^b^ (points), mean (SD)			3.0 (0.8)

^a^MMSE: Mini-Mental State Exam.

^b^TechPH: novel scale assessing older people’s attitude towards technology. The score range is 1-5. Higher scores indicate higher level of technophilia.

### Testing Issues

Testing issues are summarized in Table 2. Excluding failure due to technical or physical issues impairing the ability to complete a test, failure to complete a test due to misunderstanding of instructions was observed for 11 patients (10 four-finger–tapping test, 2 one-finger–tapping, and 1 block design test). Age, gender, education level, MMSE, and TechPH scores were not associated with failure. The patient in the cohort with the lowest MMSE (21 points) misunderstood instructions in both the four- and one-finger–tapping tests.

**Table 2:** Testing issues identified by the examiner.

Test	Issue
Four-finger tapping	Of the 36 patients, 15 (42%) did not perform the test as intended because of physical difficulties (rheumatoid arthritis, fractures, or arthrosis) or misunderstanding of instructions; 3 patients did not comprehend how to use the keypad and instead tried to perform the test on the screen of the tablet.
Stroop test	A yellow button was perceived as light green by 2 patients.
Block design test	One patient did not understand when the test started.
Geriatric Depression Scale	Two patients gave a wrong answer to 1 question. Once answered it was not possible to correct.
One-finger tapping	One patient performed all tests with the left hand. One patient needed extra instructions from the examiner regarding that the buttons were supposed to be tapped and not to drag the finger between buttons.

### Technical Issues

We observed 2 technical issues. In 3 test sessions of the 10-word list test, 1 instruction was repeated several times. For 1 testing session, 2 pdf reports were created.

## Discussion

### Principal Findings

We developed CoGNIT, a tablet app for assessing cognitive function in several cognitive domains in an automatic, standardized, and efficient manner. A previous version was developed for assessment of hydrocephalus patients, which has now been redesigned for tablet computers and expanded with new tests for more general assessments, aimed at aiding diagnostics and tracking of cognitive function in patients at memory clinics. This study aimed to assess the feasibility of testing patients with symptoms of MCI.

Overall, the feasibility for testing with patients with symptoms of MCI was good, although some issues were identified. Software bugs were identified that are straightforward to correct. Some tests did not perform optimally. On a group level, there was no indication that this was due to a lower level of a technophilia personality trait or cognitive ability as measured by the TechPH and MMSE scales. However, cognitive ability might interfere with testing in the lower range of the MMSE, as the patient with the lowest MMSE in the cohort failed to complete 2 of the included tests due to miscomprehension of instructions.

Most notably, 42% of the patients did not perform the four-finger–tapping test as intended, both due to physical limitations (eg, rheumatoid arthritis) and difficulty understanding instructions. Though a measure of manual dexterity, scores from this test correlate with tests of executive function [11]. Tests of executive function have a component of “getting it,” and this test might just be too cognitively demanding for the MCI population. Also, introducing a second means of input from the attached keyboard might be confusing. The test was originally included in the CoGNIT battery because of evidence for assessing patients with hydrocephalus [12]. A more prevalent disease in memory clinics is any of the parkinsonian syndromes, where the one-finger–tapping test has evidence [3,13]. After evaluation, the four-finger–tapping test was discarded in favor of this test in further deployment of CoGNIT.

Other testing issues are more straightforward to improve. The block design and the one-finger–tapping tests will be improved with updated instructions for the tests. In the Stroop test, a simple color adjustment will be done to improve discrimination between yellow and green. In the GDS, a button to access the previous question will be added.

### Limitations

Though inclusion criteria at the first visit included an MMSE score in the range of 20 to 28, many patients scored higher at the second visit, when the CoGNIT was administered. There was a skewed distribution, with many patients scoring in the higher range. The feasibility for testing with more cognitively impaired patients is thus less tested. However, sensitive neuropsychological testing is needed less in the cognitive range where screening instruments show marked impairment.

### Comparison With Prior Work

There are several computerized test batteries for neuropsychological testing of older adults [14]. Most batteries are administered by a trained testing technician who explains instructions for each test. This hampers standardization and scalability. A major criticism of computerized tests has been a lack of reports of reliability, normative data, validity, and, finally, poorly designed computer-person interfaces. All these issues were addressed when designing CoGNIT. Also, testing free recall memory is, to our knowledge, unique to CoGNIT. Free recall is the most sensitive test for disorders of episodic memory. We believe this will give CoGNIT an edge in early detection of cognitive disorders.

### Conclusions

The feasibility for automatic neuropsychological testing with the CoGNIT app on a tablet device in patients with symptoms of MCI was good. After minor modifications, the app is ready for further studies. The next step is collecting normative data from a healthy population.

## Abbreviations

BTH: Blekinge Institute of Technology
GDS: Geriatric Depression Scale
MCI: mild cognitive impairment
MMSE: Mini-Mental State Exam
